# Power-Up for Mucoadhesiveness: Two Generations of Thiolated Surfactants for Enhanced Sticky Nanoemulsions

**DOI:** 10.1021/acsbiomaterials.3c01207

**Published:** 2023-11-23

**Authors:** Dennis To, Gergely Kali, Soheil Haddadzadegan, Arne Matteo Jörgensen, Katharina Nigl, Fabrizio Ricci, Andreas Bernkop-Schnürch

**Affiliations:** † Center for Chemistry and Biomedicine, Department of Pharmaceutical Technology, Institute of Pharmacy, University of Innsbruck, Innrain 80/82, 6020 Innsbruck, Austria; ‡ Thiomatrix Forschungs- und Beratungs GmbH, Trientlgasse 65, 6020 Innsbruck, Austria

**Keywords:** thiolation, S-protection, surfactant, nanoemulsion, mucoadhesive

## Abstract

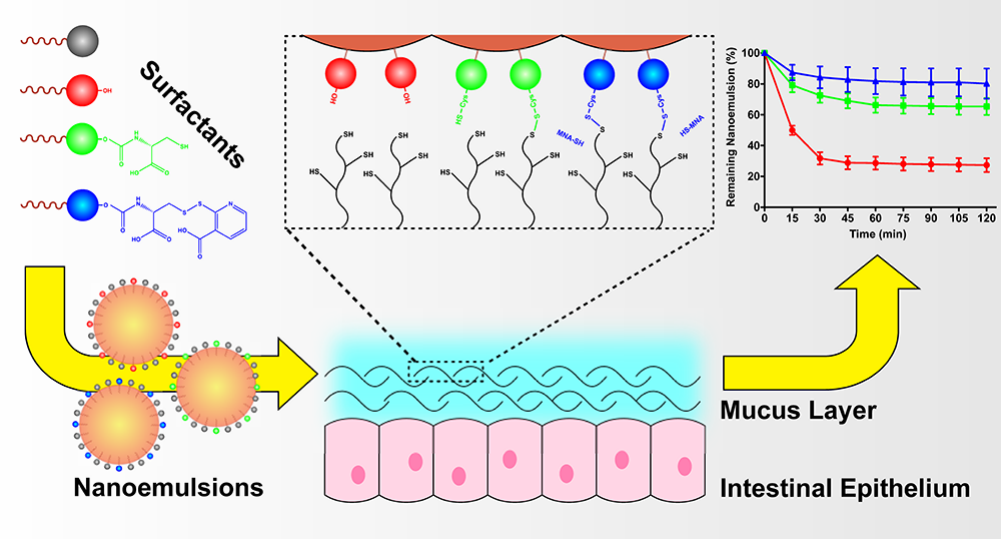

Nanoemulsions can be tuned toward enhanced gastro-intestinal retention time by incorporating thiolated surfactants into their surface. Tailoring the chemical reactivity of the thiol headgroup has major influence on mucoadhesive features of the nanoemulsion. Two generations of thiolated surfactants were synthetically derived from PEG-40-stearate featuring either a free thiol group or an S-protected thiol group. The surfactants were characterized regarding critical micelle concentration (CMC), hemolytic activity, and cytotoxicity. Subsequently, they were incorporated into nanoemulsions and the resulting nanoemulsions were characterized regarding particle size, polydispersity index (PDI), zeta potential, and time-dependent stability. Afterward, mucosal interactions as well as mucoadhesion on porcine intestinal mucosa were investigated. Successful synthesis of Cysteine-PEG-40-stearate (CYS-PEG-40-stearate) and MNA-Cysteine-PEG-40-stearate (MNA-CYS-PEG-40-stearate) was confirmed by ^1^H NMR spectroscopy. Both chemical modifications led to slightly elevated CMC values while preserving low cytotoxicity and hemotoxicity. Incorporation into nanoemulsions had minor influence on overall physical particle characteristics, while interactions with mucus and mucoadhesiveness of the nanoemulsions were drastically improved resulting in the rank order PEG-40-stearate < CYS-PEG-40-stearate < MNA-CYS-PEG-40-stearate. Accordingly, thiolated surfactants, especially S-protected derivatives, are versatile tools to generate highly mucoadhesive nanoemulsions.

## 1 Introduction

A vast number of newly discovered chemical entities is poorly water-soluble, resulting in low oral bioavailability.^1^ On the other hand, some drugs that exhibit sufficient water solubility, such as peptides, are either enzymatically or chemically degraded by the harsh pH conditions in the gastro-intestinal tract.^2,3^ In the recent past, lipid-based nanocarriers such as nanoemulsions emerged as a sound solution to overcome both problems.^3−5^ Nanoemulsions are simply formed by emulsifying an oily phase, in which the drug is dissolved, through the addition of surfactants in an aqueous phase. The formed nanoparticles allow efficient delivery of the encapsulated drug toward the gastro-intestinal absorption membrane while circumventing solubility problems and dodging degradation. To enable efficient encapsulation into nanoemulsions, drugs generally must be highly lipophilic. However, via hydrophobic ion pairing even highly hydrophilic peptides can be incorporated into lipid-based formulations as was demonstrated in previous works.^6−8^

Nonetheless, common nanoemulsions still suffer from a couple of drawbacks, one of them being rapid elimination from the gastro-intestinal tract that results in poor absorption of the incorporated drug. It has been shown that thiolation is a powerful way of enhancing the mucoadhesive properties of a system. The formation of disulfide bonds between incorporated thiol groups and cysteine-rich mucosal glycoproteins results in increased gastro-intestinal retention time, thereby enhancing oral bioavailability.^9,10^ In previous studies, covalent thiolation was mostly applied to polymers, creating so-called thiomers. While polymers of the first generation of thiomers contain free, unprotected thiol groups, the second generation of thiomers is characterized by S-protected thiol groups that are stable toward oxidation but can still form new disulfide bonds with endogenous thiols via thiol/disulfide exchange reactions.^11^ S-protected thiol groups can optionally exhibit high chemical reactivity, resulting in accelerated interaction with mucosal glycoproteins.^12−14^

In the recent past, the first efforts were made to apply thiolation to lipid-based nanoparticle formulations. As it seems pivotal to anchor the thiol functionality directly to the surface of the nanocarrier,^15,16^ the use of thiolated surfactants to cover the nanoparticle surface emerged as advantageous strategy. However, to date, the impact of incorporating thiolated surfactants with different chemical reactivity of their thiol headgroup has not been evaluated.

Thus, within this study, we aimed to compare the nanoparticle characteristics, especially mucoadhesive features, of nanoemulsions that were fabricated with two different generations of thiolated surfactants. Therefore, we synthetically derived a first generation and a second generation thiolated surfactant from the nonionic surfactant PEG-40-stearate. The surfactants were characterized regarding critical micelle concentration (CMC), cytotoxicity, and hemolytic activity. Afterward, the thiolated and nonthiolated surfactants were incorporated into nanoemulsions and the impact on particle size, polydispersity index (PDI) and zeta potential was assessed. Subsequently, interactions with mucus and the extent of mucoadhesion on porcine intestinal mucosa were investigated for each nanoemulsion.

## 2 Materials and Methods

### 2.1 Materials

PEG-40-stearate (trade name: Myrj 52) was supplied by Croda International, United Kingdom. PEG-10-oleyl ether (trade name: Brij O10), calcium chloride, coumarin-6, l-cysteine, deuterated dimethyl sulfoxide (DMSO-*d*_6_, 99.9 atom % D), dichloromethane, fetal bovine serum (FBS), 2-(4-(2-hydroxyethyl)-1-piperazinyl)-ethanesulfonic acid (HEPES), hydrogen peroxide solution (H_2_O_2_, 30% V/V), isopropylmyristate, 2-mercaptonicotinic acid (MNA), minimum essential medium (MEM), 4-nitrophenylchloroformiate, potassium chloride, resazurin sodium salt, sodium chloride, triethylamine, polysorbate 80 (trade name: Tween 80), and Triton X-100 were purchased from Sigma-Aldrich, Austria. Dithiothreitol (DTT), glucose anhydrous, Spectrum Spectra/Por dialysis tubes (cutoff: 1 kDa), *N*,*N*-dimethylformamide (DMF), and Opti-MEM were obtained from Thermo Fisher Scientific, Germany. Erythrocyte concentrate was a kind gift from Tirol Kliniken, Austria. Diethyl ether was obtained from VWR Chemicals, Austria. Ethanol was supplied by Donauchem GmbH, Austria. Penicillin/streptomycin solution was purchased from Pan-Biotech, Germany. All other reagents were of analytical grade and were obtained from commercial sources.

### 2.2 Synthesis of Two Generations of Thiolated Surfactants

#### 2.2.1 First Generation: Cysteine-PEG-40-Stearate (CYS-PEG-40-Stearate)

Synthesis of CYS-PEG-40-stearate was performed in a two-step reaction according to a previously published protocol with minor adjustments.^17^ Briefly, 1 g of unmodified PEG-40-stearate (0.5 mmol) was dissolved in 20 mL of dichloromethane, and 0.12 mL of triethylamine (0.85 mmol) was added. Then, 0.14 g of 4-nitrophenyl chloroformiate (0.70 mmol) was dissolved in 1 mL of dichloromethane and added dropwise to the mixture while cooling in an ice bath. The reaction mixture was stirred for 24 h at room temperature. Afterward, dichloromethane was evaporated by a rotavapor, and the product was lyophilized. In the second step, the solid, yellowish nitrophenyl carbonyl end-capped PEG-40-stearate and 0.15 g of l-cysteine (1.22 mmol) were dissolved in 10 mL of acetic acid-buffered saline pH 5. The reaction was initiated by increasing the pH of the mixture to 7.5–8 by the addition of 0.5 M NaOH. The mixture was stirred for 2 h at room temperature, and then the pH was adjusted back to 5 by the addition of diluted acetic acid. The resulting solution was exhaustively dialyzed with a dialysis bag (Spectrum Spectra/Por dialysis tube cutoff: 1 kDa) against 0.72 mM HCl to remove nonreacted l-cysteine. After subsequent filtration, the product was lyophilized. Afterward, a reduction with DTT was performed to eliminate disulfide bonds that may have been formed during the reaction process. Therefore, the obtained solid was dissolved in DMF and DTT was added. The reaction mixture was incubated at room temperature overnight while stirring. The next day, the mixture was precipitated in ice-cold diethyl ether and subsequently centrifuged for 10 min at 10,000 rpm. The resulting precipitate was washed multiple times with diethyl ether. Afterward, the product was lyophilized.

#### 2.2.2 Second Generation: Mercaptonicotinic Acid-Cysteine-PEG-40-Stearate (MNA-CYS-PEG-40-Stearate)

Synthesis of the S-protected derivative MNA-CYS-PEG-40-stearate was achieved by a method described by Haddadzadegan et al. for the synthesis of S-protected cyclodextrin.^13^ Briefly, MNA was oxidized to the MNA dimer, 2,2-dithiodinicotinic acid, by reacting 1 g of MNA with 1.325 mL of H_2_O_2_ (30%) at a pH of 8 in 12.5 mL of demineralized water. The reaction was incubated for 2 h at room temperature. Subsequently, the solution was diluted to a final volume of 25 mL with demineralized water. Afterward, 0.5 g of CYS-PEG-40-stearate was dissolved in demineralized water and the pH was adjusted to 8. 0.5 mL of the oxidized MNA solution was added and the reaction was stirred for 4 h at room temperature. Finally, the reaction mixture was exhaustively dialyzed with a dialysis bag (cutoff: 1 kDa) against demineralized water and subsequently lyophilized.

### 2.3 Analytical Characterization of Synthesized Surfactants

^1^H NMR measurements were performed on a “Mars” 400 MHz Avance 4 Neo spectrometer from Bruker Corporation (Billerica, MA, 400 MHz) in DMSO-*d*_6_ solution. The chemical shifts were reported in parts per million, and the center of the deuterated solvent, DMSO-*d*_6_, served as the internal standard (δ 2.5 ppm).

Additionally, FT-IR spectra of each compound were recorded using a Spectrum Two spectrometer (PerkinElmer, Beaconsfield, United Kingdom). The displayed spectra are the mean of four scans measured from 4000 to 400 cm^–1^ at a resolution of 1 cm^–1^. To allow a direct comparison of the recorded spectra, transmission values were normalized to 100% by OriginPro 2020.

### 2.4 Determination of Critical Micelle Concentration

The CMCs of the synthesized surfactants and unmodified PEG-40-stearate were determined by drop shape analysis with a drop shape analyzer (Kruess DSA25E, Kruess GmbH, Hamburg, Germany) as previously described.^18,19^ Therefore, surfactant solutions were prepared in several concentrations between 2 and 0.02 mg/mL in demineralized water. Each solution was filled into a syringe and dropwise analyzed at 25 °C using the pendant drop technique. A recommended B-value (0.4–0.6) computed by the software (Advance 1.4.2, Kruess GmbH, Hamburg, Germany) allowed correct adjustment of the drop volume. The corresponding surface tension was obtained and plotted against the concentration of the surfactant by using OriginPro 2020. A piecewise linear fit function was used to determine the CMC of each surfactant as the intersection point of two linear extrapolations (flat and steep segment) applied to the respective surface tension plot.

### 2.5 Evaluation of Hemotoxicity

A hemolysis assay was carried out to evaluate potential blood toxicity of the synthesized surfactants compared with unmodified PEG-40-stearate.^20^ Therefore, surfactant solutions were prepared in concentrations of 2, 1, 0.2, 0.1, and 0.02 mg/mL in HEPES-buffered saline pH 7.4 (HBS, containing 1 g/L glucose, 20 mM HEPES, 5 mM KCl, 136.7 mM NaCl, and 1 mM CaCl_2_). If necessary, samples were ultrasonicated to achieve complete dissolution of the surfactants. HBS served as negative control, while Triton X-100 0.1% (m/V) in HBS was used as positive control. Prior to the experiment, erythrocyte concentrate was diluted 1:200 with HBS. Afterward, 250 μL of surfactant solutions were mixed with 250 μL of diluted erythrocytes and incubated for 24 h at 37 °C in a shaking incubator at 100 rpm. Samples were subsequently centrifuged at 500 g and 100 μL supernatant of each sample were withdrawn. The supernatants were added to a 96-well plate and the absorbance was measured at 415 nm. Hemolysis was calculated according to eq 1:
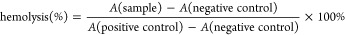
1

### 2.6 Evaluation of Cytotoxicity on the Caco-2 Cell Line

Cytotoxicity of the surfactants was assayed on the Caco-2 cell line (ECACC 86010202).^12,21^ Cells were seeded in a sterile 96-well cell culture plate at a concentration of 2 × 10^4^ cells per well with a final volume of 100 μL of MEM supplemented with 10% (V/V) heat-inactivated FBS and penicillin/streptomycin solution (100 units/0.1 mg/L)). The cells were incubated for 3 days at 37 °C in an atmosphere of 95% relative humidity and 5% CO_2_ to reach a confluent monolayer. Surfactant solutions were prepared in concentrations of 1, 0.5, 0.1, 0.05, and 0.01 mg/mL in Opti-MEM. If necessary, samples were ultrasonicated to achieve complete dissolution of the surfactants. A Triton X-100 solution 0.1% (m/V) in Opti-MEM served as negative control while Opti-MEM served as positive control for cell viability. After discarding remaining MEM supernatant, 100 μL of each sample was added to the wells. The samples were incubated for 24 h on the cells at 37 °C. After the incubation period, the supernatant was discarded and 150 μL of an 8.8 μM resazurin solution were added. Cells were incubated for another 2 h, before 100 μL of the supernatant of each well was transferred to a 96-well fluorescence plate, and fluorescence intensity was measured at an excitation wavelength of 540 nm and an emission wavelength of 590 nm with the plate reader. Cell viability was calculated according to Equation 2:
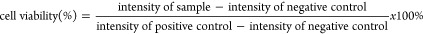
2

### 2.7 Development of Thiolated and Nonthiolated Nanoemulsions

A screening was carried out to find suitable excipients for the formation of nanoemulsions with thiolated and nonthiolated PEG-40-stearate. First, 2 mg of each surfactant was dissolved in 10 mL of demineralized water. After complete dissolution, cosurfactants and oily phase were added to reach a concentration of 0.2% (m/V) for the emulsion. The mixtures were vortexed and afterward incubated at 25 °C, 1000 rpm for 30 min on a thermomixer (Thermomixer, Eppendorf, Germany). Subsequently, particle size, PDI, and zeta potential were determined by dynamic and electrophoretic light scattering with a Zetasizer (Zetasizer Nano ZSP, Malvern Panalytical, UK).

### 2.8 Stability Study

A stability study was carried out to evaluate sufficient stability of the developed nanoemulsion at body temperature in the presence of electrolytes.^22^ Therefore, nanoemulsions prepared according to the protocol described above were subsequently diluted 1:1 with HBS. Afterward, the emulsions were incubated on a Thermomixer at 37 °C and 500 rpm. Size, PDI and zeta potential of the nanoemulsions were evaluated at the beginning of the experiment, as well as after 3, 6, and 24 h of incubation by dynamic and electrophoretic light scattering with a Zetasizer.

### 2.9 Purification of Mucus

Harvesting and purification of porcine intestinal mucus was carried out as previously described.^12^ Porcine small intestine excised from freshly slaughtered pigs was provided by a local slaughterhouse. It was cut into pieces of approximately 10 cm and opened longitudinally. Parts containing chyme were discarded. Subsequently, mucus was gently collected from the mucosa by scraping it with a finger. The harvested, unpurified mucus was diluted with 0.1 M NaCl solution in a ratio of 1:5 (m:V) and slowly stirred for 1 h under cooling. Afterward, the mixture was centrifuged at 10 °C and 10,400 g for 2 h, and the resulting supernatant as well as granular particles on the bottom of the tube were discarded. The remaining mucus was diluted with 0.1 M NaCl solution in a ratio of 1:2.5 (m:V), followed by stirring for 1 h and centrifugation as described above. Finally, the supernatant was discarded, and the purified mucus was frozen at −20 °C for further experiments.

### 2.10 Rheological Measurements

Interactions between thiolated and nonthiolated nanoemulsions with mucus were investigated by time-dependent rheological measurements with a cone–plate combination rheometer (HAAKE Mars Rheometer, Thermo Scientific, Vienna, Austria).^13,16^ Therefore, 100 μL of nanoemulsions prepared in 50 mM HEPES buffer at pH 6.8 in a concentration of 0.2% (m/V) were mechanically mixed with 500 mg of mucus in a Petri dish. Samples were either directly transferred to the lower plate of the rheometer (0 h value) or after 2 or 4 h of additional incubation at 37 °C. Subsequently, the linear viscoelastic region of the samples was determined by oscillatory strain sweep measurements at 37 °C at a frequency of 1 Hz in a range of 0.01–50 Pa. The dynamic viscosity was recorded by using the HAAKE Rheo Win 3 software.

### 2.11 Mucoadhesion Study

Mucoadhesive properties of the prepared thiolated and nonthiolated nanoemulsions were evaluated by a setup that was previously described in the literature with slight modifications.^13,23^ Briefly, porcine intestinal mucosa, obtained from a local slaughterhouse, was cut into 3 cm × 2 cm segments and mounted onto a half-cut 50 mL Falcon tube. The tube was fixed with double-sided adhesive tape in an angle of 45° in an incubator providing 37 °C and 100% relative humidity. A peristaltic pump set at 0.5 mL/min was used to continuously rinse the mucosa with 50 mM HEPES buffer, pH 6.8, mimicking intestinal pH conditions. Prior to the experiment, each piece of mucosa was rinsed for 10 min. Nanoemulsions were prepared in 50 mM HEPES buffer pH 6.8 in a concentration of 0.2% (m/V) and labeled with Coumarin-6 0.1% (m/m). A 100 μL aliquot of each formulation was applied to the mucosa and equilibrated horizontally for 20 min. For the blank, no sample was applied. Subsequently, the pump was started, and each mucosa was rinsed for a period of 120 min. Every 15 min, the washoff was collected in an empty Falcon tube and centrifuged for 10 min at 10,500 rpm. A 100% control was prepared by adding 100 μL of the nanoemulsions to 7.5 mL of the supernatant collected from the initial washing step.

Afterward, 1 mL of the supernatant of each Falcon Tube was transferred to an Eppendorf tube containing 1 mL of ethanol, mixed, and incubated for 2 h at 37 °C in a shaking incubator. Finally, samples were centrifuged at 13,400 rpm for 10 min, and 100 μL of each tube was transferred to a black fluorescence plate and fluorescence intensity was measured at an excitation wavelength of 445 nm and emission wavelength of 510 nm. The amount of nanoemulsion remaining on the mucosa at each time point was calculated using eq 3:
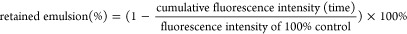
3

### 2.12 Statistical Data Analysis

Statistical data analysis was performed using one-way ANOVA in combination with a Bonferroni post hoc-test to analyze the significance of differences between means of more than two groups calculated with GraphPad Prism 5.01. The level of *p* < 0.05 was set as the minimum level of significance.

## 3 Results and Discussion

### 3.1 Synthesis of CYS-PEG-40-Stearate and MNA-CYS-PEG-40-Stearate

**Figure 1:**
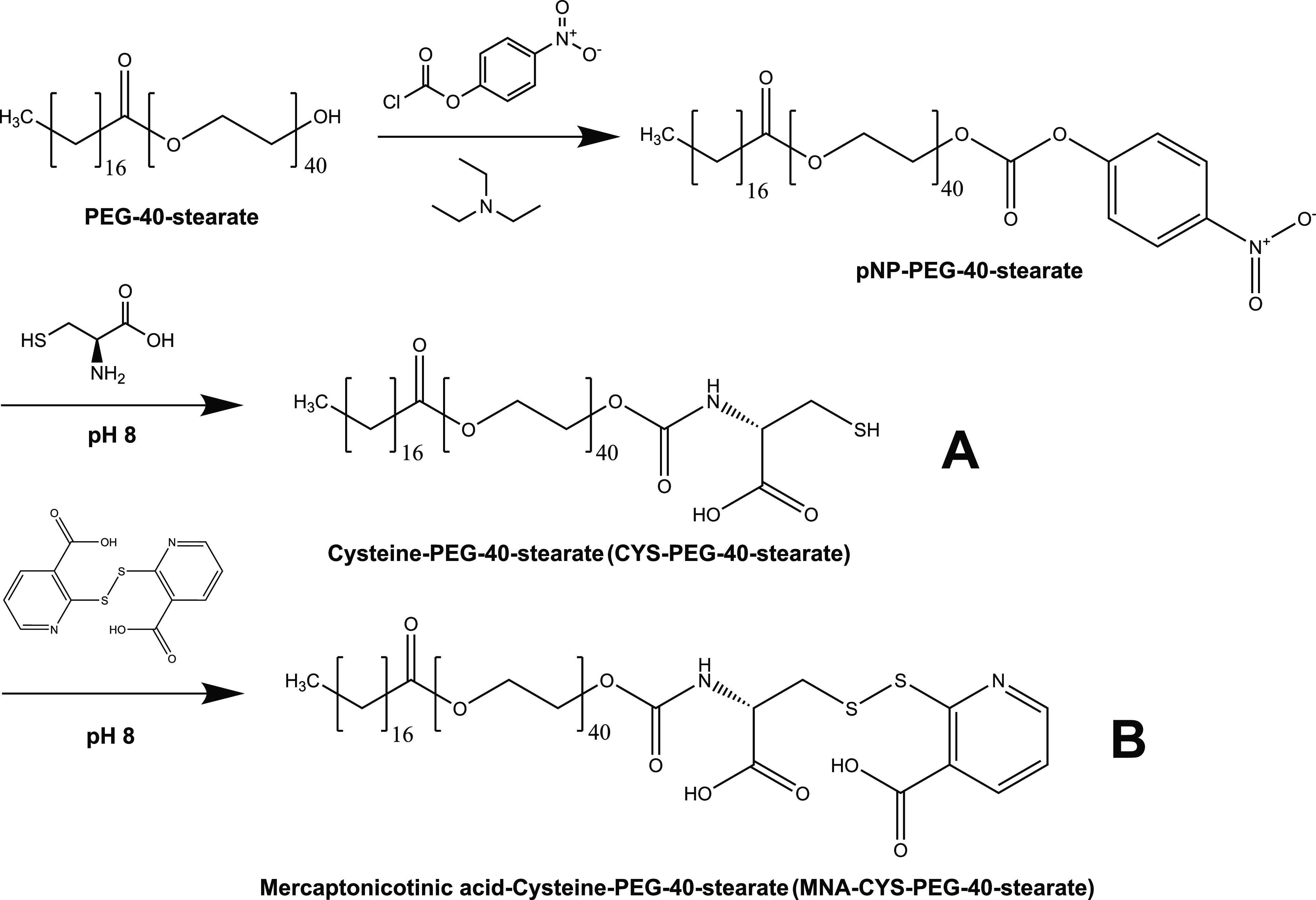
Synthetic pathway for CYS-PEG-40-stearate (A) and MNA-CYS-PEG-40-stearate (B).

The synthetic pathway for both thiolated surfactants is illustrated in Figure 1. CYS-PEG-40-stearate was synthesized in an approximate yield of 20%. However, additional workup with DTT significantly decreased the yield afterward. Accordingly, the synthesis was repeated to acquire enough starting material for the synthesis of the second-generation thiolated surfactant. MNA-CYS-PEG-40-stearate was obtained with an approximate yield of 80% by reacting CYS-PEG-40-stearate with dimeric MNA. Corresponding NMR spectra are depicted in Figure S1. They confirmed that both products were successfully formed. Additionally, FT-IR spectra of unmodified and modified PEG-40-stearate were recorded and are shown in Figure S2. However, a comparison of the three FT-IR traces showed only minor differences.

### 3.2 Influence of Thiolation on CMC of PEG-40-Stearate

**Figure 2:**
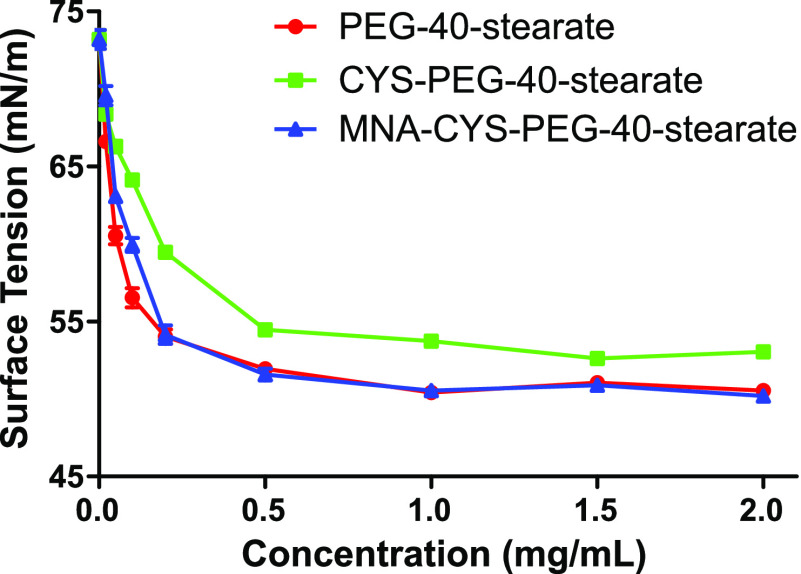
Surface tension plots of PEG-40-stearate (red circles), CYS-PEG-40-stearate (green squares), and MNA-CYS-PEG-40-stearate (blue triangles) in the range 0–2 mg/mL. Data are the means ± standard deviation of at least three experiments. A piecewise linear fit function was used to determine the CMC as the intersection point of two linear extrapolations applied to each graph.

The CMC is a crucial inherent property of each surfactant and marks the concentration above which micelles are formed.^24^ As these aggregates might behave differently compared to the monomeric surfactant in solution, the CMC value possibly has major influence on traits such as surfactant toxicity and solubilizing properties as well as the suitability to be applied in nanoparticular delivery systems. Therefore, it was of great interest to evaluate the possible impact of the performed chemical modifications on the CMC of the native PEG-40-stearate. Figure 2 shows the plots of surface tension versus concentration of each surfactant in demineralized water at 25 °C. The CMC can be read at the intersection point of two linear extrapolations (the steep and the flat segment of the curve) applied to each plot. Accordingly, the CMC of unmodified PEG-40-stearate was determined to be 0.11 mg/mL.

Literature values reported on this surfactant are in the range of 0.11–0.63 mg/mL^25−27^ and vary mainly because of the different method of determination, ambient temperature, and the use of different media to dissolve the surfactant. Nonetheless, drop shape analysis appeared to be a suitable method for experimental determination of the CMC of the investigated surfactants. The CMC values calculated for the synthetic derivatives CYS-PEG-40-stearate and MNA-CYS-PEG-40-stearate are 0.27 and 0.14 mg/mL, respectively. Both CMCs are thus slightly higher but in a similar range as the initial CMC of the unmodified surfactant. Especially, the surface tension plot of MNA-CYS-PEG-40-stearate showed hardly any difference from the curve of PEG-40-stearate.

### 3.3 Toxicological Characterization

**Figure 3:**
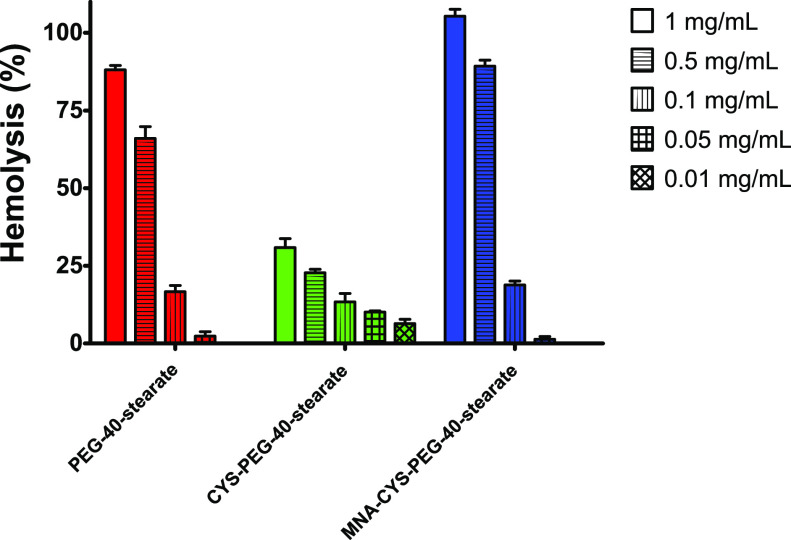
Hemolytic activity of PEG-40-stearate (red), CYS-PEG-40-stearate (green), and MNA-CYS-PEG-40-stearate (blue) on human erythrocytes over 24 h in the concentrations 1 mg/mL (bars), 0.5 mg/mL (horizontal striped bars), 0.1 mg/mL (vertical striped bars), 0.05 mg/mL (grid bars), and 0.01 mg/mL (diamond bars). Data are means ± standard deviation of at least three experiments.

Hemolytic activities of the synthesized surfactants in comparison to unmodified PEG-40-stearate are illustrated in Figure 3.

To evaluate a possible impact of the altered CMC value for each surfactant, we chose concentrations above the CMC, in the range of the CMC and below the CMC. In the case of unmodified PEG-40-stearate, the resulting hemolysis increased very significantly in the concentration range between 0.1 and 0.5 mg/mL (17% to 66%), which seems to be in coincidence with the CMC value of approximately 0.11 mg/mL. Increased solubilization of the erythrocyte membrane by micelles could be a reasonable explanation for the strongly elevated hemolysis levels observed for concentrations ≥0.5 mg/mL of PEG-40-stearate. A similar behavior in this concentration range was observed for MNA-CYS-PEG-40-stearate. In this case, the elevation in hemolysis was even more pronounced (19% to 89%). According, to Manaargadoo-Catin et al., there are two types of surfactant-mediated hemolysis, namely, osmotic way lysis and membrane solubilization. While osmotic way lysis is mainly induced by monomeric surfactants interacting with the cellular membrane of the erythrocytes, membrane solubilization requires the presence of surfactant micelles.^28^ Taking the results of CMC determination into account, it is therefore likely that monomeric PEG-40-stearate-based surfactant solutions are lowly hemolytic while micelle formation tremendously increases their hemolytic activity due to membrane solubilzation. However, hemolysis of the first generation thiolated surfactant CYS-PEG-40-stearate does not exceed 31% throughout the whole concentration range, presenting the lowest hemolytic activity of the investigated surfactants. A possible reason might be the CMC value of CYS-PEG-40-stearate, which is 0.27 mg/mL and therefore around 2-fold higher than that of PEG-40-stearate and MNA-CYS-PEG-40-stearate. This surfactant has a weaker tendency to form micelles, thereby reducing hemolysis by membrane solubilization.

**Figure 4:**
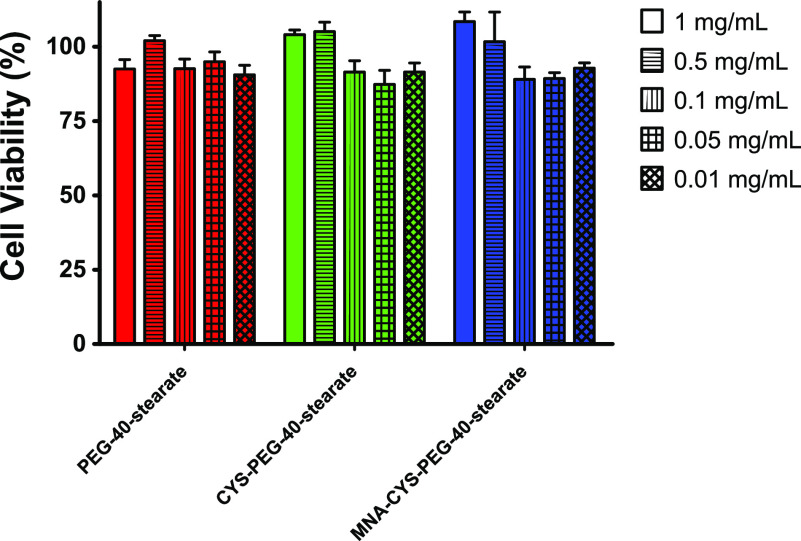
Viability of the Caco-2 cell line after 24 h of incubation with solutions of PEG-40-stearate (red), CYS-PEG-40-stearate (green), and MNA-CYS-PEG-40-stearate (blue) in the concentrations 1 mg/mL (bars), 0.5 mg/mL (horizontal striped bars), 0.1 mg/mL (vertical striped bars), 0.05 mg/mL (grid bars), and 0.01 mg/mL (diamond bars). Data are means ± standard deviation of at least three experiments.

Results of the cytotoxicity assay on the Caco-2 cell line are summarized in Figure 4. All compounds were well tolerated by the cells resulting in cell viabilities ≥85% throughout the whole concentration range. No dose-dependent toxicities were observed, which is in contrast to the results obtained by the hemolysis assay. Accordingly, the formation of micelles is likely leading to more pronounced interaction with the cell membrane, as seen in the hemolytic activity, but does not directly cause cellular death. Thus, it can be concluded that the thiolation of PEG-40-stearate did not have a negative impact on cytotoxic potential of the surfactant in both cases.

Interestingly, charged surfactants typically exhibit higher cytotoxicity than nonionic surfactants resulting in the rank order cationic > anionic > amphoteric > nonionic.^29^ However, in this case, the introduction of anionic charges by the incorporation of cysteine and MNA did not lead to an apparent increase in cytotoxicity. This could possibly be attributed to the overall huge molecular weight of the surfactants, which exceeds 2 kDa. Hence, the impact of a minor number of additional anionic charges on the cytotoxic potential of the surfactant molecule is likely relatively low.

### 3.4 Formation and Characterization of the Nanoemulsions

A preliminary screening was carried out to find a suitable nanoemulsion formulation that allows incorporation of nonthiolated as well as thiolated PEG-40-stearate. The final compositions are depicted in Table 1 (top). The amount of surfactant in each formulation was constantly kept at 10% (m/m) in relation to the total weight of nonaqueous components. Besides exchanging PEG-40-stearate with CYS-PEG-40-stearate or MNA-CYS-PEG-40-stearate respectively, all other compounds of the formulation were kept identical to allow direct comparison of the impact of the exchanged surfactant only. Respective particle sizes, PDI, and zeta potential of the three formulations in a concentration of 0.2% (m/V) are summarized in Table 1 (bottom).

**Table 1:** Composition of Developed Nanoemulsions (Top); Particle Size, PDI, and Zeta Potential of Nanoemulsions in a Concentration of 0.2% (m/V) (Bottom)^a^

	PEG-10-oleyl ether	polysorbate 80	isopropylmyristate	PEG-40-stearate	CYS-PEG-40-stearate	MNA-CYS-PEG-40-stearate	demineralized water
F1	6 mg	4 mg	8 mg	2 mg			10 mL
F2	6 mg	4 mg	8 mg		2 mg		10 mL
F3	6 mg	4 mg	8 mg			2 mg	10 mL
		Size (nm)		PDI		Zeta potential (mV)	
F1		108.9 ± 22.0		0.23 ± 0.02		–11.6 ± 1.25	
F2		113.2 ± 19.5		0.24 ± 0.04		–21.3 ± 1.55	
F3		99.9 ± 5.8		0.25 ± 0.10		–18.6 ± 9.55	

a Data are means ± standard deviation of at least three experiments.

All formulations were capable of spontaneous self-emulsification by simply mechanical mixing. Additional ultrasonication to decrease the particle size and improve emulsification was not required. The nanoemulsions exhibited a particle size of around 100 nm and a PDI below 0.3. Incorporation of chemically modified surfactants did not significantly alter the physical particle characteristics. However, a slight influence on zeta potential was observed, as it decreased from initially −11.6 mV for unmodified PEG-40-stearate to −21.3 and −18.6 mV for CYS-PEG-40-stearate and MNA-CYS-PEG-40-stearate, respectively. This is in good agreement with the molecular structures of the thiolated surfactants, as both of them are pH-dependent negatively charged.

Subsequently, a stability study was carried out to investigate potential instabilities of the nanoemulsions in the presence of electrolytes. The results are summarized in Table 2. All formulations showed only minor changes in the particle size distribution after 6 h, confirming short-term stability. However, after 24 h, significant increases in particle size as well as PDI were observed, which is pointing toward limited long-term stability. Among the formulations, F1 appeared to be least stable as the measured average particle size increased to more than 1000 nm with a PDI of 0.7 over 24 h, indicating a polydisperse size distribution. F2 and F3 were likely less instable than F1 because of their more negative surface charge, favoring particle repulsion, and hindering agglomeration.^30^ Yet, stability of the emulsions can be considered sufficient for further experiments.

**Table 2:** Particle Size and PDI of Nanoemulsions after 1:1 Dilution with HBS over 24 h of Incubation^a^

	0 h	3 h	6 h	24 h
F1 size (nm)	143.5 ± 20.0	125.9 ± 1.2	110.6 ± 1.6	1097.4 ± 226.7
F1 PDI	0.27 ± 0.07	0.25 ± 0.01	0.17 ± 0.00	0.73 ± 0.16
F2 size (nm)	136.7 ± 14.3	146.8 ± 10.9	96.39 ± 1.1	363.9 ± 79.6
F2 PDI	0.22 ± 0.03	0.27 ± 0.06	0.25 ± 0.01	0.31 ± 0.09
F3 size (nm)	106.4 ± 5.0	113.7 ± 17.7	85.5 ± 9.6	364.8 ± 105.7
F3 PDI	0.24 ± 0.02	0.21 ± 0.06	0.30 ± 0.04	0.37 ± 0.05

a Data are means ± standard deviation of at least three experiments.

### 3.5 Rheological Investigations

**Figure 5:**
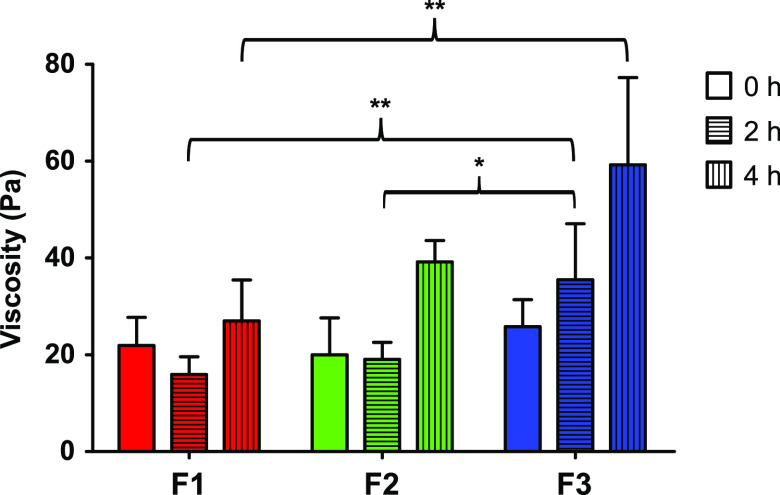
Viscosity of mucus incubated with 0.2% (m/V) of the nanoemulsions F1 (red), F2 (green), and F3 (blue) in a ratio of 5:1 (m:V) after 0 h (bars), 2 h (horizontal striped bars), and 4 h (vertical striped bars). Data are means ± standard deviation of at least three experiments. Significant differences are indicated as *p < 0.05 and **p < 0.01.

Rheological measurements were carried out to investigate the potential interactions of the developed nanoemulsions with mucus. Thiolated excipients are able to form disulfide bonds with cysteine-rich mucus glycoproteins, thereby causing an increase in viscosity. Strong interactions between nanocarrier and mucus should thus be mirrored by an apparent time-dependent increase in viscosity.^13,16^ Results of the rheological studies are illustrated in Figure 5.

While incubation of the control nanoemulsion F1 with mucus did not lead to a significant change in viscosity, both thiolated nanoemulsions exhibited time-dependent increase in viscosity. After 4 h of incubation with mucus, the viscosity of mucus mixed with formulations F2 and F3 increased 2.0 and 2.3-fold compared to their initial values. Moreover, mucus mixed with F3 showed at each time point a higher viscosity than F1 and F2, reaching a maximum of 59 Pa after 4 h. The results highlight the influence of S-protection on the reaction kinetics of the thiolated surfactants with mucosal glycoproteins. The disulfide group in the second generation thiolated surfactant MNA-CYS-PEG-40-stearate yields higher reactivity toward disulfide exchange reactions with mucosal glycoproteins than the free thiol group of the first generation thiolated surfactant CYS-PEG-40-stearate. Thus, the highly interactive surface of nanoemulsion F3 facilitates pronounced mucosal interactions, leading to a faster and also a higher increase in observed mucus viscosity.

### 3.6 Mucoadhesion

**Figure 6:**
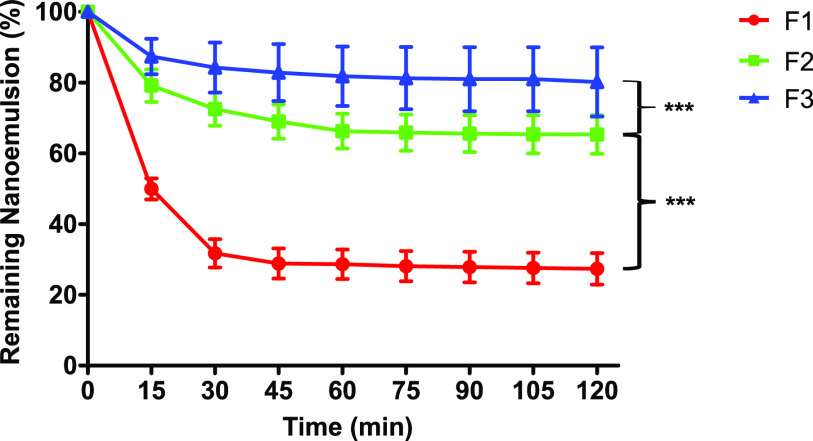
Mucoadhesion of the nanoemulsions F1 (red circles), F2 (green squares), and F3 (blue triangles) on porcine intestinal mucosa. The fluorescent marker coumarin-6 was used to label each emulsion in a concentration of 0.1% (m/m). Data are means ± standard deviation of at least three experiments. Significant differences are indicated as ***p < 0.001.

Formulations exhibiting increased mucoadhesiveness have the potential to prolong the retention time of an incorporated drug in the intestine. This can be highly beneficial for oral drug delivery, potentially leading to a higher bioavailability. As depicted in Figure 6, the choice between thiolated or nonthiolated PEG-40-stearate drastically changes mucoadhesive properties of the formulation. The nonthiolated nanoemulsion was eliminated to a significantly higher extent as well as faster from the intestinal mucosa compared to the thiolated nanoemulsions. After 2 h, around 27% of the unmodified nanoemulsion was retained on the mucosa while the thiolated nanoemulsions F2 and F3 showed 65% and 80% retention, respectively. This corresponds overall to a 2.4- and a 3.0-fold improvement in mucoadhesion in relation to the control nanoemulsion. Furthermore, MNA-CYS-PEG-40-stearate demonstrated significantly higher mucosal retention (*p* < 0.001) compared to CYS-PEG-40-stearate, confirming superiority of the second generation of thiolation versus the first generation. This is in good agreement with the results of the rheological investigations, in which the magnitude of mucosal interactions increased in the rank order F1 < F2 < F3. Considering the fact, that the PEG-40-stearate-based surfactants comprised only about 10% of the nanoemulsions, while all other excipients used in F1–F3 were exactly identical, the impact of the thiolated surface on the mucoadhesive behavior of the nanoemulsion is tremendous.

## 4 Conclusions

Thiolated mucoadhesive drug delivery systems are designed to increase the gastrointestinal retention time and bioavailability of drugs by facilitating interactions with mucosal glycoproteins via a disulfide reaction. Besides the more renowned thiolated polymers, lipid-based nanoparticular drug delivery systems, such as SLN, NLC, or nanoemulsions, can also be tuned toward increased mucoadhesiveness.^14,16^ In this case, especially the design of the surface plays a crucial role for interaction of the nanoparticles with the gastrointestinal environment.^15^ The surfactants described in this work represent two different types of thiolated surfactants, featuring either a free thiol group (first generation) or an S-protected thiol group (second generation). To the best of our knowledge, it was the first time that two generations of thiolated surfactants were synthesized, incorporated into nanoemulsions and subjected to a direct comparison of their mucoadhesive features. Both synthesized surfactants exhibited CMC values in the range of unmodified PEG-40-stearate and the chemical modification did neither negatively impact on hemotoxic potential nor on cytotoxicity. They were successfully incorporated into nanoemulsions with only minor influence on particle size, PDI, and zeta potential compared to a nanoemulsion produced with the unmodified PEG-40-stearate. However, mucosal interactions of the emulsions were heavily influenced by the choice of surfactant and increased in the rank order unmodified < first generation < second generation as depicted in the rheological investigations. In good agreement with this, *ex vivo* mucoadhesion experiments on porcine intestinal mucosa resulted in the same rank order with the second generation on top. This is especially impressive considering that in each case the thiolated surfactant comprised only about 10% of the formulation. Accordingly, the application of different generations of thiolated surfactants in nanoemulsions is an efficient way of altering nanoparticular surface characteristics and represents a powerful tool to obtain pronounced mucoadhesive nanoemulsions.

## Supporting Information Available

NMR and IR spectra of synthesized surfactants (PDF)
