# Supplementary material for: Power-Up for Mucoadhesiveness: Two Generations of Thiolated Surfactants for Enhanced Sticky Nanoemulsions
Source: ACS Biomater Sci Eng. 2023 Nov 23;9(12):6797–804. doi: 10.1021/acsbiomaterials.3c01207 (PMC10716821; doi:10.1021/acsbiomaterials.3c01207)
Supplement: Supplementary file 1 — ab3c01207_si_001.pdf [file ab3c01207_si_001.pdf]

Power-Up for Mucoadhesion: Two generations of thiolated surfactants for enhanced sticky nanoemulsions

Supplementary Material (4 Pages, 2 Figures)

Dennis To[1], Gergely Kali[1], Soheil Haddadzadegan[1], Arne Matteo Jörgensen[1], Katharina Nigl[1], Fabrizio Ricci[2] and Andreas Bernkop-Schnürch[1*]

[1]*Center for Chemistry and Biomedicine, Department of Pharmaceutical Technology, Institute of Pharmacy, University of Innsbruck, Innrain 80/82, 6020 Innsbruck, Austria*

[2]*Thiomatrix Forschungs- und Beratungs GmbH, Trientlgasse 65, 6020 Innsbruck, Austria*

---

[*]Corresponding author:

Center for Chemistry and Biomedicine, Department of Pharmaceutical Technology, Institute of Pharmacy, University of Innsbruck, Innrain 80/82, 6020 Innsbruck, Austria

Tel. +43 512 507 58 600

Email: Andreas.Bernkop@uibk.ac.at

NMR spectra are depicted in **Figure S1** and confirm successful synthesis of CYS-PEG-40-stearate (1st generation, **A**) and MNA-CYS-PEG-40-stearate (2nd generation, **B**).

CYS-PEG-40-stearate: ^{1}H NMR (DMSO-d_6, 400 MHz) δ/ppm = 6.70-6.60 (broad m, -N*H*-) 4.56 (s, -C*H*<), 4.110 (s, -CH_2-C(O)-), 3.51 (s, -CH_2- PEG backbone), 2.82-2.67 (m, -CH_2-SH), 2.28 and 1.51 (s, -CH_2-CH_2-C(O)-O- stearyl), 1.23 (s, -CH_2- stearyl), 0.85 (s, -CH_3).

MNA-CYS-PEG-40-stearate: ^{1}H NMR (DMSO-d_6, 400 MHz) δ/ppm = 8.40, 7.80, 7.40 (s, aromatic -C*H*=), 6.73-6.45 (broad m, -N*H*-) 4.56 (s, -C*H*<), 4.110 (s, -CH_2-C(O)-), 3.51 (s, -CH_2- PEG backbone), 2.82-2.67 (m, -CH_2-SH), 2.28 and 1.51 (s, -CH_2-CH_2-C(O)-O- stearyl), 1.23 (s, -CH_2- stearyl), 0.85 (s, -CH_3).

Additionally, FT-IR spectra of the synthesized compounds were recorded and compared with unmodified PEG-40-stearate, as illustrated in **Figure S2**. No significant differences between the three FT-IR spectra were found. This can likely be attributed to the fact, that the chemical modifications performed are minor in relation to the total size of the molecule backbone, thus hardly influencing FT-IR results.

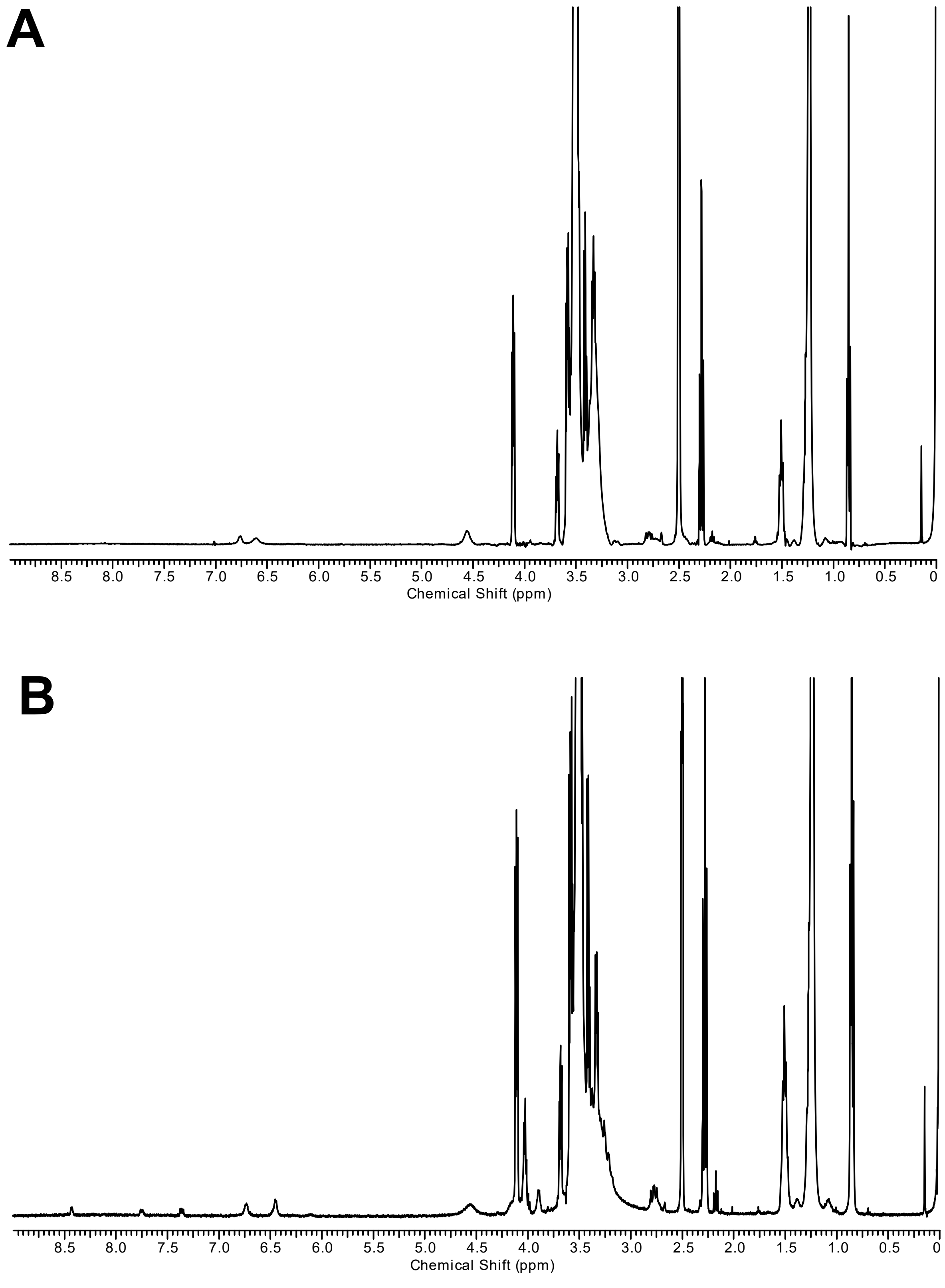

Figure S1. ^{1}H-NMR spectra (400 MHz) of CYS-PEG-40-stearate (**A**) and MNA-CYS-PEG-40-stearate (**B**) in DMSO-d_6.

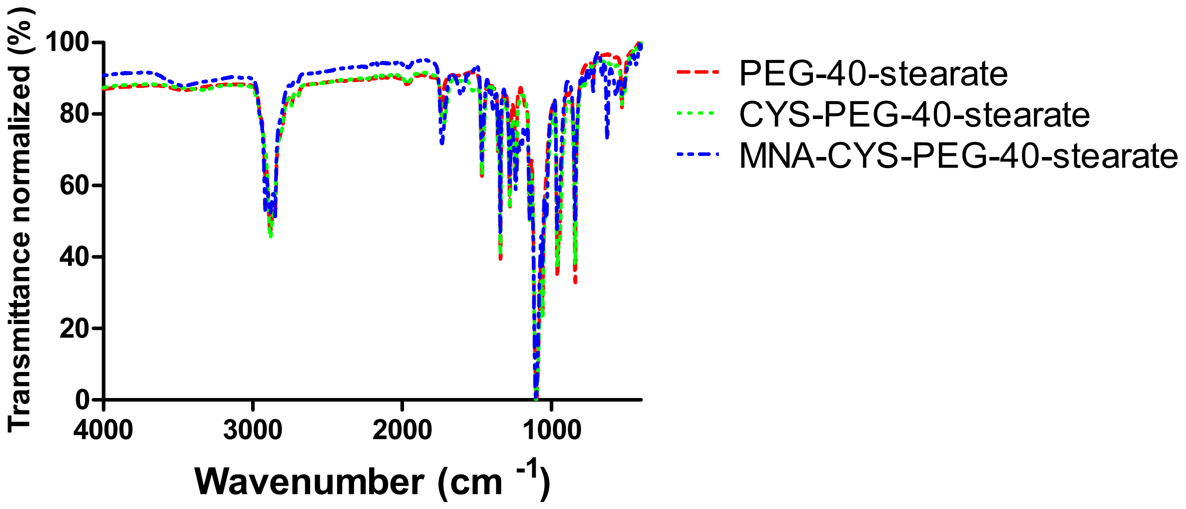

Figure S2. FT-IR spectra of PEG-40-stearate (red), CYS-PEG-40-stearate (green) and MNA-CYS-PEG-40-stearate (blue). Transmittance values were normalized by OriginPro 2020.
